# *Tm*Atg6 Plays an Important Role in Anti-Microbial Defense Against *Listeria monocytogenes* in the Mealworm, *Tenebrio molitor*

**DOI:** 10.3390/ijms21041232

**Published:** 2020-02-12

**Authors:** Tariku Tesfaye Edosa, Yong Hun Jo, Maryam Keshavarz, Ki Beom Park, Jun Ho Cho, Young Min Bae, Bobae Kim, Yong Seok Lee, Yeon Soo Han

**Affiliations:** 1 Department of Applied Biology, Institute of Environmentally-Friendly Agriculture (IEFA), College of Agriculture and Life Sciences, Chonnam National University, Gwangju 61186, Korea; bunchk.2000@gmail.com (T.T.E.); yhun1228@jnu.ac.kr (Y.H.J.); mariakeshavarz1990@gmail.com (M.K.); misson112@naver.com (K.B.P.); junhojo12@naver.com (J.H.C.); ugisaka@naver.com (Y.M.B.); kbb941013@gmail.com (B.K.); 2 Department of Life Science and Biotechnology, College of Natural Sciences, Soonchunhyang University, Asan City 31538, Korea; yslee@sch.ac.kr

**Keywords:** *TmAtg6*, induction pattern, autophagy, intracellular bacteria, RNAi

## Abstract

Autophagy-related gene-6 (Beclin-1 in mammals) plays a pivotal role in autophagy and is involved in autophagosome formation and autolysosome maturation. In this study, we identified and characterized the autophagy-related gene-6 from *Tenebrio molitor* (*TmAtg6*) and analyzed its functional role in the survival of the insect against infection. The expression of *TmAtg6* was studied using qRT-PCR for the assessment of the transcript levels at various developmental stages in the different tissues. The results showed that *TmAtg6* was highly expressed at the 6-day-old pupal stage. Tissue-specific expression studies revealed that *TmAtg6* was highly expressed in the hemocytes of late larvae. The induction patterns of *TmAtg6* in different tissues of *T. molitor* larvae were analyzed by injecting *Escherichia coli*, *Staphylococcus aureus*, *Listeria monocytogenes*, or *Candida albicans*. The intracellular Gram-positive bacteria, *L. monocytogenes*, solely induced the expression of *TmAtg6* in hemocytes at 9 h-post-injection, whilst in the fat body and gut, bimodal expression times were observed. RNAi-mediated knockdown of the *TmAtg6* transcripts, followed by a challenge with microbes, showed a significant reduction in larval survival rate against *L. monocytogenes*. Taken together, our results suggest that *Tm*Atg6 plays an essential role in anti-microbial defense against intracellular bacteria.

## 1. Introduction

Macroautophagy (autophagy) collectively refers to a group of intracellular degradation pathways that mediate the breakdown of intracellular material in lysosomes [1,2]. It is an evolutionarily conserved, catabolic process associated with multiple biological and physiological processes. Besides, it performs protective and defensive functions with respect to innate immunity, inflammation, and resistance against microbial infection [3,4].

The process of autophagy extends from autophagosome formation to the degradation of non-self, damaged, or surplus cell components by lysosomal hydrolases via a series of steps [5,6]. In selective autophagy, the specific cargos are first tagged by ubiquitination and recognized by the autophagy adaptor molecules for subsequent targeting to autophagosomes for degradation [7]. The selective autophagy mainly targets misfolded proteins, damaged organelles, and intracellular pathogens like *Mycobacterium* spp., *Salmonella* spp., and *Listeria* spp. [8]. Based on the cargo being delivered for degradation, xenophagy is the cargo that contains intracellular pathogens [8]. Therefore, in xenophagy, pathogen-containing phagosomes are exclusively targeted for autophagic degradation [9]. In either no-selective or selective autophagy, various autophagy-related genes are involved in different stages of autophagy (initiation, phagophore formation, elongation, and completion) [10].

In yeast, where the majority of the molecular mechanisms of autophagy have been studied, the Atg1 kinase complex with Atg13 and Atg17 induces membrane isolation and initiates autophagy [11,12]. The phosphatidylinositol (PtdIns) 3-kinase complex (complex I: Vps15-Vps34-Vps30/ Atg6-Atg14; and complex-II: Vps15-Vps34-Vps30/Atg6-Atg14-Vps38) [13,14] and Atg9 complex [15] affect phosphoinositides to recruit proteins for phagophore and autophagosome formation. Finally, the ubiquitin-like protein conjugation complexes (Atg8, Atg12, Atg4, Atg7, and Atg3, among other proteins) elongate and complete the autophagy process [16]. The roles of *Tm*Atg3, *Tm*Atg5, and *Tm*Atg8 in mediating the autophagy-based clearance of *Listeria* in *T. molitor* have been investigated previously [17,18].

Atg6 (Beclin-1 in mammals) is highly conserved between yeast and mammals. It plays a pivotal role in autophagy and is involved in autophagosome formation and autolysosome maturation by forming a complex with Vps34, Vps15, UVRAG, and Vps38 [19].

Atg6 is relatively unique since it is not only autophagy-specific, but also has different functions; for example, the Atg6/Vps30 complex is required for autophagy, sorting vacuolar contents [20], and pollen germination [21], and acts as a tumor suppressor gene [22,23]. Therefore, biologically, Atg6 is required for life span extension in both animals and plants in supplying the cells with energy under adverse conditions, maintaining critical levels of metabolism, and clearing microbial infections. However, the molecular function of Atg6 in *T. molitor* has not yet been studied. Thus, we sought to identify the function of Atg6 in *T. molitor* with respect to the molecular mechanism of autophagy during microbial infection in beetles. Therefore, in this study, we characterized the role of *TmAtg6* in insect survivability against bacterial challenge using RNAi gene silencing. We demonstrated that *TmAtg6* has a specific molecular function in the immune response against the intracellular bacteria, *L. monocytogenes.*

## 2. Results

### 2.1. Sequence Identification and In Silico Analysis of TmAtg6

**Figure 1:**
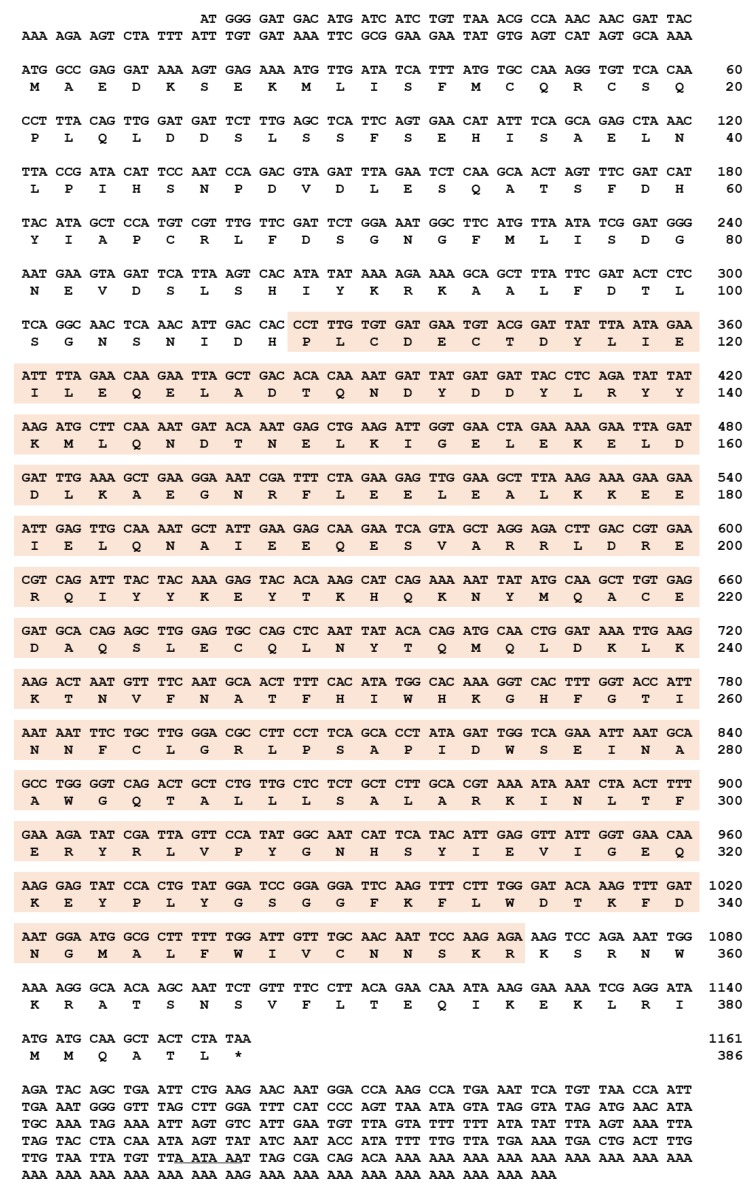
Nucleotide and deduced amino acid sequences of *TmAtg6*. The full-length open reading frame (ORF) sequence of *TmAtg6* gene was identified. *TmAtg6* has 1161 bp of ORF encoding 386 amino acid (aa) residues. Domain analysis indicates that *Tm*Atg6 contains one Atg6 domain. The polyadenylation signal sequence (AATAA) is underlined in the 3′-UTR region and the *TmAtg6* domain is shaded in orange.

**Figure 2:**
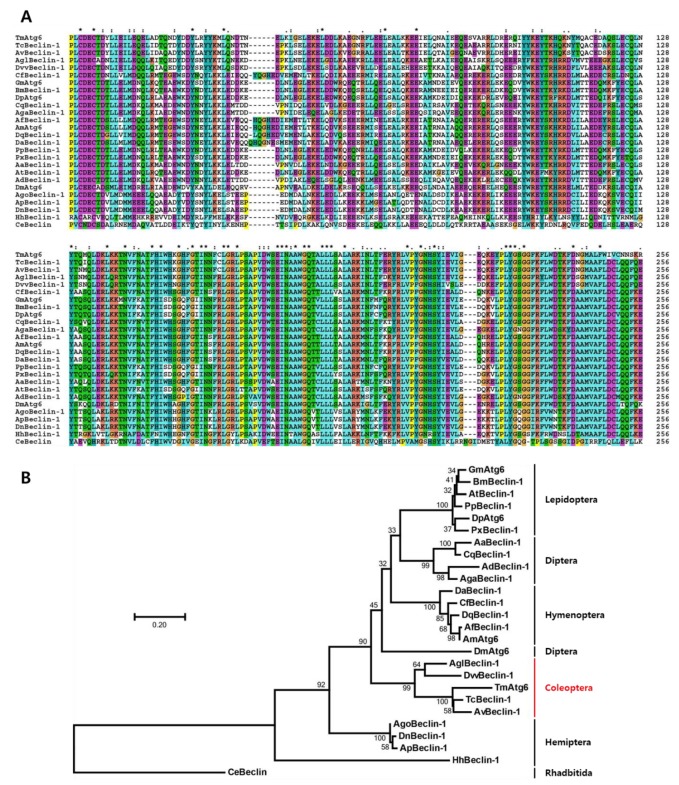
Multiple alignments and molecular phylogenetic analysis of insect beclin-1 homologs protein. (**A**) Multiple sequence alignment of *Tm*Atg6 along with its homologs. The highly conserved beclin-1 domain was aligned by using Clustal X2 software. The symbols indicate conservation scores between groups according to the Gonnet PAM 250 matrix (‘*’ > ‘:’ > ‘.’) and ‘−’ indicates internal or terminal gaps. (**B**) Phylogenetic analyses of *Tm*Atg6 homologs were performed based on the multiple alignments using the Clustal X2 and the phylogenic tree was constructed by MEGA7 programs using the maximum likelihood and bootstrapped of 1000 replications. The red color text with vertical line used to indicate the coleopteran order grouped together. Phylogenetic analysis of the *Caenorhabditis elegans beclin-1* sequence was used as the outgroup. The following protein sequences were used to construct the phylogenetic tree. *Tm*Atg6 (*Tenebrio molitor* Autophagy-related 6), *Tc*Beclin-1 (*Tribolium castaneum* Beclin-1-like protein; EFA06871.2), *Av*Beclin-1 (*Asbolus verrucosus* beclin-1-like protein; RZC37309.1), *Agl*Beclin-1 (*Anoplophora glabripennis* beclin-1-like protein; XP_018570978.1), *Dvv*Beclin-1 (*Diabrotica virgifera* beclin-1-like protein; XP_028144478.1), *Ago*Beclin-1 (*Aphis gossypii* beclin-1-like protein; XP_027849435.1), *Ap*Beclin-1 (*Acyrthosiphon pisum* beclin-1-like protein; XP_016661093.1), *Hh*Beclin-1 (*Halyomorpha halys* beclin-1-like protein; XP_014275231.1), *Dn*Beclin-1 (*Diuraphis noxia* PREDICTED: beclin-1-like protein; XP_015372132.1), *Aa*Beclin-1 (*Aedes aegypti* AAEL010427-PA; EAT37604.1)’ *Ad*Beclin-1 (*Anopheles darling* beclin-1; ETN63629.1), *Ag*aBeclin-1 (*Anopheles gambiae* str. PEST AGAP003858-PA; EAA06006.4), *Cq*Beclin-1 (*Culex quinquefasciatus* beclin-1 EDS35627.1), *Dm*Atg6 (*Drosophila melanogaster* Autophagy-related 6; AAF56227.1), *Dp*Atg6 (*Danaus plexippus* autophagy related protein Atg6; EHJ78273.1), *Gm*Atg6 (*Galleria mellonella* autophagy related protein Atg6; AFP66875.1), *Pp*Beclin-1 (*Papilio polytes* beclin-1-like protein; XP_013145468.1), *Px*Beclin-1 (*Plutella xylostella* beclin-1-like protein; XP_011559638.1), *Bm*Beclin-1 (*Bombyx mori* autophagy related protein Atg6; ACJ46062.1), *At*Beclin-1 (*Amyelois transitella* beclin-1-like protein XP_013199196.1), *Af*Beclin-1 (*Apis florea* beclin-1-like protein isoform X1; XP_003696795.1), *Am*Atg6 (*Apis mellifera* autophagy specific gene 6 isoform X2; XP_392365.1), *Da*Beclin-1 (*Diachasma alloeum* beclin-1-like protein isoform X1; XP_015116372.1), *Cf*Beclin-1 (*Camponotus floridanus* beclin-1-like protein isoform X1; XP_011257589.1), *Dq*Beclin-1 (*Dinoponera quadriceps* beclin-1-like protein isoform X2; XP_014479707.1), *Ce*Beclin (*Caenorhabditis elegans* Beclin (human autophagy) homolog; CCD62215.1).

Full-length cDNA sequence of *TmAtg6* was obtained from the *T. molitor* RNAseq database and 5′- / 3′-RACE PCR. *TmAtg6* contains an open reading frame (ORF) of 1161 bp that encodes a protein of 386 amino acid residues (Figure 1). The 5′- and 3′-untranslated regions (UTR) of *TmAtg6* were 105 and 348 bp in length, respectively. The poly (A) signal (AATAAA) was found 9 bp upstream of the poly (A) tail. The multiple alignments of *Tm*Atg6 amino acid sequence with its orthologs indicates a high degree of conservation in the Atg6 amino acid sequence within insects (Figure 2A). The phylogenetic analysis revealed Coleopteran insects (*Asbolus verrucosus* beclin-1-like protein and *Tribolium castaneum* Beclin-1-like protein) grouped together (Figure 2B). Furthermore, the percentage of identity showed that *Tm*Atg6 is closest to *Tribolium castaneum* (*Tc*Beclin-1) since they share the highest sequence identity (89%) (Figure S1).

### 2.2. Developmental and Tissue-Specific Expression Patterns of TmAtg6

**Figure 3:**
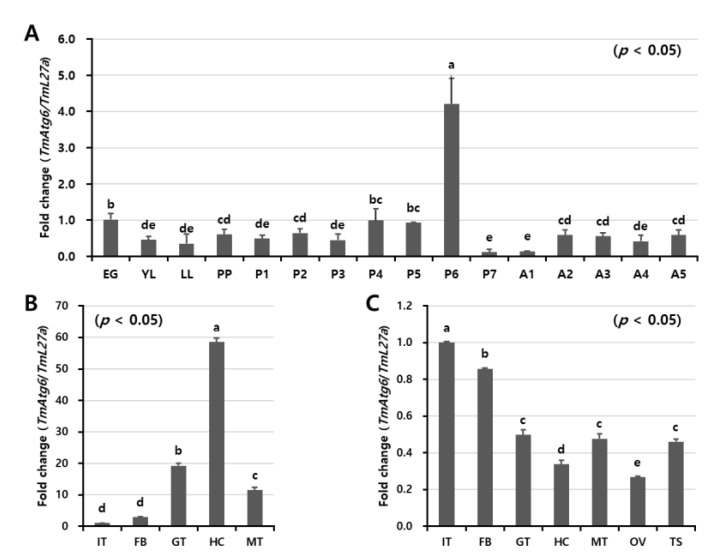
Developmental and tissue-specific expression patterns of *TmAtg6* gene. (**A**) The developmental stages of mealworm, egg (EG), young larvae (YL), late larvae (LL), pre-pupa (PP), 1–7-days old pupae (P1–P7), and 1–5-days old adult (A1–A5), were examined to study the expression level of *TmAtg6*. For each stage, 20 individuals were used to extract RNA with the subsequent synthesis of cDNA. The results indicate that *TmAtg6* expression was gradually increased from young larvae to 2-days old pupae with highest expression at the 6-days old pupal stage. In adult stages, there was no considerable expression difference. Tissue-specific expression patterns of *TmAtg6* genes in late larvae (**B**) and in 5-day-old adults (**C**). Hemocytes, gut, fat body, Malpighian tubules, and integument (for late instar larvae and adults), and testes and ovaries (for adults) were dissected and collected from a total of 20 late larvae and 5-day-old adults. The results indicate that *TmAtg6* was highly expressed in hemocytes, while low expression was observed in the integument and fat body in late larvae. In adults, the expression levels of *TmAtg6* were high in fat body and integument. IT; integument, GT; gut, FB; fat body, HC; hemocytes, MT; Malpighian tubules, OV; ovary, and TS; testis. *Tenebrio* ribosomal protein 27a (*TmL27a*) was used as internal control.

The expression levels of *TmAtg6* transcripts at different developmental stages and tissues were analyzed by qRT-PCR (Figure 3A). *TmAtg6* transcripts were detected through all developmental stages and in all examined tissues. The highest expression level was observed in pupal stages, specifically in six-day-old pupae. Comparatively lower *TmAtg6* transcript levels were observed in young and late instar larvae, 7-day-old pupae, and 1-day-old adults.

In the tissues, the highest *TmAtg6* expression levels were observed in the hemocytes of late larvae and the integument and fat body of adults (Figure 3B,C). Conversely, *TmAtg6* expression levels were low in the integument of late larvae and the hemocytes of adults.

### 2.3. Temporal Induction Pattern of TmAtg6

**Figure 4:**
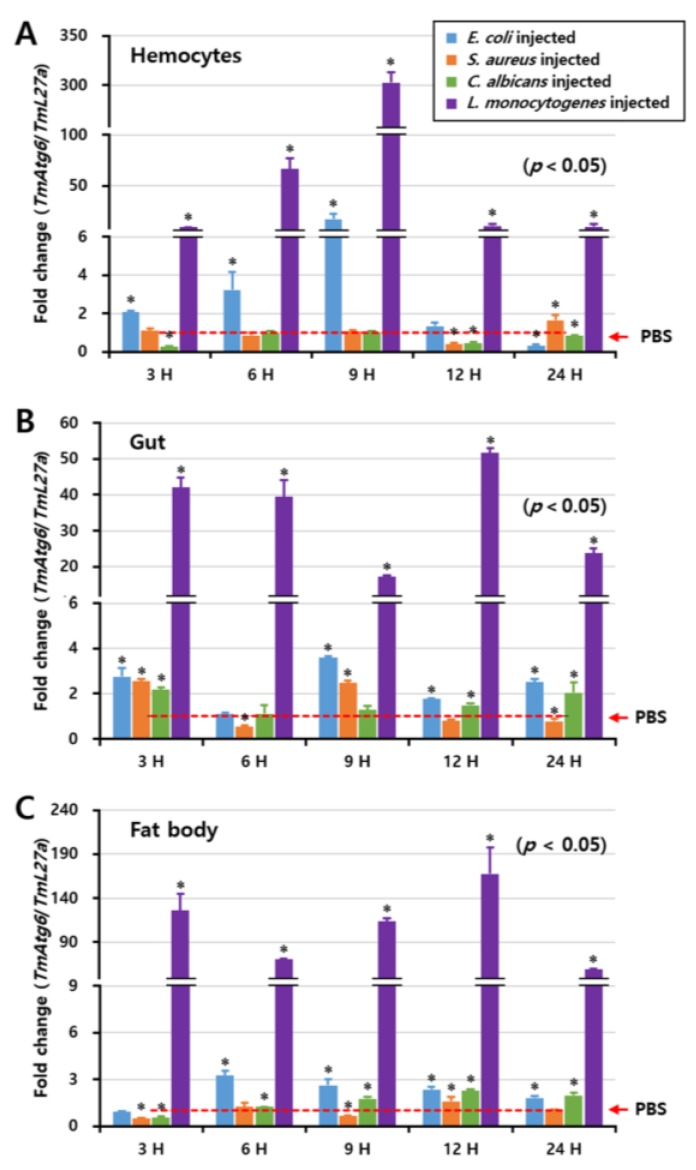
Induction patterns of *TmAtg6* in different tissues against *E. coli*, *S. aureus*, *C. albicans*, and *L. monocytogenes*, including hemocytes (**A**), gut (**B**), and fat body (**C**). The induction pattern analysis of *TmAtg6* gene in different tissues of *T. molitor* young larvae was performed by injection of *E. coli* (10^6^ cells/μL), *S. aureus* (10^6^ cells/μL), *C. albicans* (5 × 10^4^ cells/μL), or *L. monocytogenes* (10^6^ cells/μL). Samples were collected at different time points such as 3, 6, 9, 12, and 24 h post-injection of microorganisms. Twenty young larvae of mealworm were used at each time point. In hemocytes, *TmAtg6* gene was highly induced at 9 h post-injection of *L. monocytogenes*. In the gut, the injection of *L. monocytogenes* highly induced the expression of *TmAtg6* at 3 h post-injection, gradually decreased during 6 and 9 h, and then highly induced levels of *TmAtg6* 12 h post-injection. In the fat body, injection of *L. monocytogenes* highly induced *TmAtg6* at 12 h post-infection.

To investigate the inducibility of the *TmAtg6* gene, *E. coli*, *S. aureus*, *L. monocytogenes*, or *C. albicans* were injected into *T. molitor* larvae and the three immune tissues such as hemocytes, fat body, and gut were time-dependently (at 3, 6, 9, 12, and 24 h post-injection) collected for the analysis of the induction pattern by qRT-PCR. Remarkably, *TmAtg6* mRNA was highly induced by *L. monocytogenes* in all tissues (Figure 4), whereas *E. coli*, *S. aureus,* and *C. albicans* slightly induce *TmAtg6* mRNA. In hemocytes, the induction of *TmAtg6* by *L. monocytogenes* showed the highest expression at 9 h post-injection. In the gut, *TmAtg6* expression was represented in two patterns, being highly expressed at 3 h post-injection, slightly expressed at 6 and 9 h post-injection, and increased again at 12 h post-injection. In the fat body, the highest expression of *TmAtg6* was detected at 3 and 12 h post-injection of *L. monocytogenes*.

### 2.4. Effect of TmAtg6 RNAi on T. molitor Survivability 

**Figure 5:**
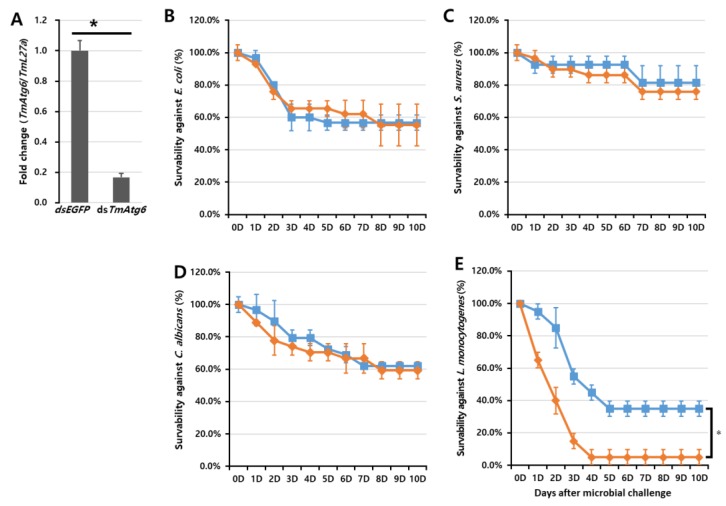
RNA interference (RNAi)-based functional study of *TmAtg6* in *Tenebrio molitor* larvae. Larval survival curves following infection with microorganisms. *TmAtg6* knockdown (**A**), *E. coli* (**B**), *S. aureus* (**C**), *C. albicans* (**D**), *L. monocytogenes* (**E**). * significant at *p* < 0.05. Results represent the average of three independent replicates with standard error. *TmAtg6* gene silencing significantly affected the survivability of *T. molitor* larvae against *L. monocytogenes* infection. In contrast, the *TmAtg6* gene silencing did not show significant differences in survivability against *E. coli*, *S. aureus*, and *C. albicans*.

To characterize the function of *TmAtg6* against microorganisms, we silenced its transcript levels using RNAi and challenged *T. molitor* larvae with prepared microorganisms. The percentage of *TmAtg6* downregulation was confirmed (84% knockdown) by qRT-PCR prior to microbial challenge. The survivability of the *TmAtg6*-silenced *T. molitor* larvae against microbial injection was monitored for 10 days. The injection of ds*TmAtg6* and/or ds*EGFP* did not affect the survival of *T. molitor* larvae when injected with the control PBS. Compared to the ds*EGFP*-injected larval group, no significant difference was detected in the ds*TmAtg6*-injected group on larval survivability against any of the tested microorganisms except *L. monocytogenes* (Figure 5). Surprisingly, *TmAtg6*-silenced larvae showed higher susceptibility against *L. monocytogenes* compared to the ds*EGFP* group.

## 3. Discussion

Autophagy 6 (Beclin 1 in mammalian), as a key regulator of autophagy [24], has many functions in intracellular processes such as signaling pathways, vacuolar protein sorting [25], endocytic trafficking, apoptosis [26], and degradation of sequestered cytoplasmic contents, including damaged organelles and pathogens [27,28]. Additionally, crosstalk between autophagy and antiviral immunity has been reported, suggesting the dual effect of autophagy as promoting the clearance of viral components and activating the immune system to produce antiviral cytokines [29]. Therefore, A*tg6* is an essential gene required for both development and host immunity. Specifically, the induction of autophagy (autophagosome formation) in mammals mainly depends on the Class III PI3K complex, comprising hVps34, Beclin-1, p150, and Atg14-like protein or ultraviolet irradiation resistance-associated gene (UVRAG) [30]. Similarly, in yeast, complex I (Vps15-Vps34-Vps30/Atg6-Atg14) and complex II (Vps15-Vps34-Vps30/Atg6-Atg14-Vps38) are required in autophagy and vascular protein sorting, respectively [13,30]. Thus, the current study was conducted to characterize the immune functions of *TmAtg6* in *T. molitor*.

During the development and metamorphosis of holometabolous insects, cell death occurs at each stage, and the mechanisms of cell debris removal are essential in many aspects [31]. Therefore, the roles of autophagy in development and the clearance of invading pathogens have been extensively investigated [32,33,34,35]. For instance, autophagy occurs during the remodeling and degeneration of various larval tissues [36,37], as well as during changes in larval instars and larva–pupa transition [36,38]. Our observations regarding *TmAgt6* expression in different developmental stages and tissues also suggest its importance in the growth and development of *T. molitor*. In particular, the highest expression of *TmAtg6* in 6-day-old pupae may suggest that autophagy occurs during metamorphosis from pupae to adults. Likewise, the function of autophagy during metamorphic transition has been well characterized in *Heliothis virescens* [39] and *Alabama argillacea* [40]. Additionally, in the silkworm, *Bombyx mori*, the expression levels of several *Atg* genes including *BmAtg1*, *-2*, *-6*, *-11*, *-12*, *-13*, and *-18* were high during molting and pupation stages when levels of the steroid hormone (20-hydroxyecdysone; 20E) were high [41]. Similarly, in *T. molitor*, the *TmAtg8*, *-5*, *-3,* and *-13* transcripts were expressed in all tissues, suggesting their importance in the cell remodeling process and development [17,18,42]. Moreover, the expression of *TmAtg6* in the hemocytes of *T. molitor* larvae suggests the function of *TmAtg6* in insect development. Hemocytes have been reported as vital components in the synthesis and transport of nutrients and hormones for proper growth and development and in wound healing by clearance of dead cells through phagocytosis and autophagy [43,44].

Studies show that autophagy plays an important role in not only developmental functions, but also in immunological functions such as bacterial, viral, and parasitoid clearance. In mosquitoes, the autophagy pathway is relevant to the replication and transmission of arboviruses [45,46,47]. However, in other insect species, it has been reported that autophagy is induced and is crucial for the innate cellular immune response against several intracellular bacterial, fungal, and viral pathogens [48,49,50]. Additionally, the involvement of granulocyte-associated autophagy in hemocytes was immunologically and morphologically studied, showing a high accumulation of autophagic vacuoles in activated hemocytes granulocytes [51]. For example, in *Drosophila*, studies on the importance of antiviral autophagy against Rift Valley fever virus and Vesicular stomatitis virus revealed that viral replication was increased in the absence of autophagy genes [52,53]. Moreover, the pattern-recognition receptor, Peptidoglycan-recognition protein LE (PGRP-LE), was identified as a recognition protein of the diaminopimelic acid-type peptidoglycan derived from Gram-negative bacteria to induce autophagy, consequently preventing the growth of *L. monocytogenes* and promoting host survivability [54]. Our current induction studies also revealed that *TmAtg6* is highly expressed in all tissues exclusively infected with *L. monocytogenes. TmAtg6* is comparatively highly expressed in hemocytes. Supporting this result, Bénédicte and his colleagues reported that autophagy targets *L. monocytogenes* during primary infection to limit the onset of early bacterial growth [49]. Interestingly, *TmAtg6* expression was particularly downregulated by *E. coli*, *S. aureus*, and *C. albicans* at different time points. Supporting our current finding, in the mammalian model, some microorganisms downregulate ATG genes to avoid antimicrobial autophagy [55]. This downregulation is achieved through the modification of phagosomes by blocking their maturation via fusion with autophagosomes [56]. Additionally, in insects, autophagy-associated genes are downregulated by Wolbachia through the suppression of the autophagic signal to prevent their elimination [57].

Moreover, we studied the function of *TmAtg6* in the defense response against microbial infection by silencing *Tm*Atg6 protein expression. The gene-silenced larval group showed significant susceptibility to *L. monocytogenes* infection, indicating that *TmAtg6* is directly involved in counteracting intracellular pathogen infection in the mealworms. The previous studies also reported that the other autophagy-related genes are involved in the defense response to *L. monocytogenes* (*TmAtg3*, *TmAtg5*, *Tmatg8*) [17,18], *E. coli*, and *S. aureaus* (*TmAtg13*) [42]. Collectively, *TmAtg6* plays an important role in the autophagy-dependent defense response against the intracellular pathogen *L. monocytogenes* in *T. molitor*.

## 4. Materials and Methods

### 4.1. Insect Rearing and Maintenance

The coleopteran insect, *Tenebrio molitor* (mealworm), was maintained at 27 ± 1 °C and 60% ± 5% relative humidity in the dark with an artificial diet prepared from 170 g of whole-wheat flour, 20 g of fried bean powder, 10 g of soy protein, 100 g of wheat bran, 200 mL of sterile water, 0.5 g of chloramphenicol, 0.5 g of sorbic acid, and 0.5 mL of propionic acid. For the experiments, the 10 to 12 instar larvae were used. To ensure uniformity in size, the larvae were separated according to their physical size using a set of laboratory test sieves (Pascall Eng. Co. Ltd, Crawley, Sussex, England).

### 4.2. Preparation of Microorganisms

The following microorganisms were used in this study: Gram-negative bacteria (*Escherichia coli* K12), Gram-positive bacteria (*Staphylococcus aureus* RN4220 and *Listeria monocytogenes*), and fungi (*Candida albicans*). The microorganisms were cultured in Luria-Bertani (LB; *E. coli* and *S. aureus*), Sabouraud dextrose (*C. albicans*), and brain heart infusion (BHI; *L. monocytogenes)* broths at 37 °C overnight and subcultured at 37 °C for 3 h. Then the microorganisms were harvested and washed 2 times by centrifugation at 3500 rpm for 10 min in phosphate-buffered saline (PBS; pH 7.0). They were then suspended in PBS and the concentrations were measured at OD_600_. Finally, 10^6^ cells/μL of *E. coli*, *S. aureus*, and *L. monocytogenes* and 5 × 10^4^ cells/μL of *C. albicans* were injected separately.

### 4.3. Identification and Cloning of Full-Length cDNA Sequence of TmAtg6

The *T. molitor Atg6* gene was identified by local-blastn analysis (National Center for Biotechnology Information) with the amino acid sequence of the *T. castaneum Atg6* gene (EFA06871.2) as the query search. The partial cDNA sequence of *TmAtg6* was obtained from *T. molitor* RNAseq database and the full-length cDNA sequence of *TmAtg6* (MN259540) was identified by 5′- and 3′-rapid amplification of cDNA end (RACE) PCR using a SMARTer RACE cDNA amplification kit (Clontech Laboratories, Mountain View, USA) according to the manufacturer’s instructions. PCR was performed using the AccuPower^®^ PyroHotStart Taq PCR PreMix (Bioneer, Deajeon, Korea) with *TmAtg6* specific primers (RACE primers included *TmAtg6* -cloning_Fw and *TmAtg6* -cloning_Rv; Table 1). PCR was carried out under the following conditions: Pre-denaturation at 95 °C for 5 min, followed by 35 cycles of denaturation at 95 °C for 30 s, annealing at 53 °C for 30 s, and extension at 72 °C for 2 min, and a final extension at 72 °C for 5 min using a MyGenie96 Thermal Block (Bioneer, Deajeon, Korea). PCR products were purified using an AccuPrep^®^ PCR Purification Kit (Bioneer, Deajeon, Korea), immediately ligated into T-Blunt vectors (Solgent, Deajeon, Korea), and transformed into DH5α competent cells according to the manufacturer’s instructions. Plasmid DNA was extracted from fully grown competent cells using an AccuPrep^®^ Nano-Plus Plasmid Extraction Kit (Bioneer, Deajeon, Korea), sequenced, and analyzed. Finally, the full-length cDNA sequence of *TmAtg6* was obtained.

### 4.4. Domain Analysis and Phylogenetic Analysis

Specific domains were analyzed using the InterProScan 5 and blastp programs [58,59]. Multiple alignments were performed with representative Atg6 protein sequences of other insects obtained from Genbank using Clustal X2 software [60]. Phylogenetic and percentage identity analyses were conducted using Clustal X2 and MEGA 7 programs [61]. The amino acid sequences of *CeBeclin* of Rhabditida were used as outgroups.

### 4.5. Expression Analysis of TmAtg6

Whole-body samples were collected from *T. molitor* (*n* = 20) at various developmental stages, including the eggs (EG), young instar larvae (YL; 10th–12th instar larvae), late instar larvae (LL; 19th–20th instar larvae), prepupae (PP), 1 to 7-day-old pupae (P1–P7), and 1 to 5-day-old adults (A1–A5). To investigate tissue-specific *TmAtg6* expression patterns, samples were collected from various tissues (*n* = 20), including the gut, hemocytes, integument, Malpighian tubules, and fat body of late instar larvae and 5-day-old adults, and the ovaries and testes of the adults. In addition, tissue-specific induction pattern analysis of the *TmAtg6* gene was performed by injecting *E. coli*, *S. aureus*, *L. monocytogenes*, or *C. albicans*. Three well-known immune tissues such as hemocytes, fat body, and gut were dissected and collected at 3, 6, 9, 12, and 24 h post-injection. Samples were collected in 500 μL of guanidine thiocyanate RNA lysis buffer (2 mL of 0.5 M EDTA, 1 mL of 1 M 2-(*N*-morpholino)ethanesulfonic acid (MES) Buffer, 17.72 g of guanidine thiocyanate, 0.58 g of sodium chloride, 0.7 mg of phenol red, 25 μL of Tween-80, 250 μL of acetic acid glacial, and 500 μL of isoamyl alcohol) and homogenized using a homogenizer (Bertin Technologies, Montigny-le-Bretonneux, France) at 7500 rpm for 20 s.

Total RNAs were extracted from the collected samples using the modified LogSpin RNA isolation method [62]. Briefly, the homogenized samples were centrifuged for 5 min at 13,000 rpm and 4 °C. The supernatant (300 μL) was transferred into a new 1.5 mL tube, mixed with 1 volume of pure ethanol, transferred into a silica spin column (Bioneer, Missouri City, Texas, USA, KA-0133-1), and centrifuged for 30 s at 13,000 rpm and 4 °C. The silica spin column was treated with DNase (Promega, Deajeon, Korea, M6101) at 25 °C for 15 min and washed with 3 M sodium acetate buffer and 80% ethanol. After drying by centrifugation for 2 min at 13,000 rpm and 4 °C, total RNAs were eluted with 30 μL of distilled water (Sigma, USA, W4502-1L). cDNAs were immediately synthesized with 2 μg of total RNAs using an AccuPower^®^ RT PreMix (Bioneer, Deajeon, Korea) and Oligo (dT) 12–18 primers on a MyGenie96 Thermal Block (Bioneer, Deajeon, Korea) according to the manufacturer’s instructions.

Quantitative real-time PCR (qRT-PCR) reactions were performed using an Exicycler™ 96 Real-Time Quantitative Thermal Block (Bioneer Company, Daejeon, Korea) with a gene-specific primers and AccuPower^®^ 2X GreenStar qPCR Master Mix (Bioneer, Deajeon, Korea) under the following conditions: Initial denaturation at 94 °C for 5 min, 45 cycles of denaturation at 9 °C for 15 s, and annealing at 60 °C for 30 s. The 2^−ΔΔCt^ method [63] was employed to analyze *TmAtg6* expression levels. *T. molitor* ribosomal protein L27a (*TmL27a*) was used as an internal control to normalize differences in template concentration between samples.

### 4.6. TmAtg6 Gene Silencing

cDNA synthesized from *T. molitor* hemocytes was amplified by semi-quantitative PCR using *TmAtg6* gene-specific primers (product size; 500 bp, listed in Table 1) conjugated with a T7 promoter sequence designed using SnapDragon software (http://www.flyrnai.org/cgi-bin/RNAi_find_primers.pl). The PCR reaction was carried out under the following conditions: Initial denaturation at 94 °C for 2 min followed by 35 cycles of denaturation at 94 °C for 30 s, annealing at 53 °C for 30 s, extension at 72 °C for 30 s, and a final extension at 72 °C for 5 min. PCR products were purified using the AccuPrep PCR Purification Kit (Bioneer Company, Daejeon, South Korea), and dsRNA was synthesized using an Ampliscribe™ T7-Flash™ Transcription Kit (Epicentre Biotechnologies, Madison, WI, USA) according to the manufacturer’s instructions. After synthesis, dsRNA was purified using 5 M ammonium acetate and precipitated by 80% ethanol. Subsequently, it was quantified using an Epoch spectrophotometer (BioTek Instruments Inc, Winooski, VT, USA). As a control, we synthesized dsRNA for enhanced green fluorescent protein (ds*TmEGFP*) and stored at −20 °C until use.

The synthesized ds*TmAtg6* was diluted to a final concentration of 1.5 µg/µL. dsRNA (1.5 µg/µL) was injected into young-instar larvae (10th–12th instars; *n* = 30) using disposable needles mounted onto a micro-applicator (Picospiritzer III Micro Dispense System, Parker Hannifin, Hollis, NH, USA). Another set of young-instar larvae (*n* = 30) were injected with equal amounts of ds*EGFP* used as a negative control. Injected larvae were maintained on an artificial diet under standard rearing conditions. *TmAtg6* knockdown was evaluated and over 90% of knockdown was achieved at 2 days post-injection.

### 4.7. Survivability Assay

*E. coli* (10^6^ cells/μL), *S. aureus* (10^6^ cells/μL), *L. monocytogenes* (5 × 10^6^ cells/μL), and *C. albicans* (5 × 10^4^ cells/μL) were prepared according to the protocol as described above. Bioassays were conducted by injecting 1.5 μg/μL ds*TmAtg6* into the hemocoel of young larvae. Two days post-injection, the knockdown level was confirmed by qRT-PCR, and prepared microorganisms were injected into both the ds*TmAtg6* and ds*EGFP*–treated larval groups. The challenged larvae were maintained, and the number of living larvae was recorded for 10 days. The survival rates of the *TmAtg6*-silenced group were compared to those of the control groups. All the experiments were triplicated. Statistical analysis was conducted using SAS 9.4 software (SAS Institute Inc., Cary, NC, USA), and the cumulative survival ratios were analyzed by Tukey’s multiple test at a significance level of *p* < 0.05.

## 5. Conclusions

In this study, we identified and characterized the immunological function of *TmAtg6* in *T. molitor*. The induction patterns of *TmAtg6* in response to microbial challenges and survivability study confirmed that the *Tm*Atg6 plays a key role against *L. monocytogenes* infection in *T. molitor*. We are now focusing on the characterization of the *Tm*Atg6-Atg14L-Vps34-Vps15 complex in autophagosome formation in mealworms during the microbial challenge.

## Figures and Tables

**Table 1:** Sequences of the primers used in this study.

Name	Primers sequence
TmAtg6_qRTPCR_Fw TmAtg6-qRTPCR-Rv	5′-AGCTCCaTGTCGTTTGTTCG-3′ 5′-GGTGGTCAATGTTTGAGTTGCC-3′
TmAtg6-T7_Fw TmAtg6-T7-Rv	5′-TAATACGACTCACTATAGGGT AGCTCCATGTCGTTTGTTCG-3′ 5′-TAATACGACTCACTATAGGGT GTCAATGTTTGAGTTGCC-3′
TmBeclin1 5′RACE GSP1 TmBeclin1 5′RACE GSP2	5′-TGTGAACACCTTTGGCACAT-3′ 5′-CACACAAAGGGTGGTCAATG-3′
TmBeclin1 3′RACE GSP1 TmBeclin1 3′RACE GSP2	5′-GAAATTGGAAAAGGGCAACA-3′ 5′-TGCTCTGTTGCTCTCTGCTC-3′
TmL27a_qPCR_Fw TmL27a_qPCR_Rv	5′-TCATCCTGAAGGCAAAGCTCCAGT-3′ 5′-AGGTTGGTTAGGCAGGCACCTTTA-3′
dsEGFP_Fw dsEGFP_Rv	5′-TAATACGACTCACTATAGGGT CGTAAACGGCCACAAGTTC-3′ 5′-TAATACGACTCACTATAGGGT TGCTCAGGTAGTGTTGTCG-3′

※ Underlines indicate T7 promoter sequences.

## Supplementary Materials

Supplementary materials can be found at https://www.mdpi.com/1422-0067/21/4/1232/s1.
