# Supplementary material for: TmAtg6 Plays an Important Role in Anti-Microbial Defense Against Listeria monocytogenes in the Mealworm, Tenebrio molitor
Source: Int J Mol Sci. 2020 Feb 12;21(4):1232. doi: 10.3390/ijms21041232 (PMC7072900; doi:10.3390/ijms21041232)
Supplement: Supplementary file 1 [file ijms-21-01232-s001.pdf]

Supplementary Figure

*Tm*Atg6 Plays an Important Role in Anti-Microbial Defense Against *Listeria monocytogenes* in the Mealworm, *Tenebrio molitor*

Tariku Tesfaye Edosa [1,†], Yong Hun Jo [1,†], Maryam Keshavarz [1], Ki Beom Park [1], Jun Ho Cho [1], Young Min Bae [1], Bobae Kim [1], Yong Seok Lee [2] and Yeon Soo Han [1,*]

1 Department of Applied Biology, Institute of Environmentally-Friendly Agriculture (IEFA), College of Agriculture and Life Sciences, Chonnam National University, Gwangju 61186, Korea
2 Department of Life Science and Biotechnology, College of Natural Sciences, Soonchunhyang University, Asan city 31538, Korea
* Correspondence: hanys@jnu.ac.kr; Tel.: +82-62-530-2072
† These authors contributed equally to this work.

	TmAtg6	TcBeclin-1	AvBeclin-1	AglBeclin-1	DvvBeclin-1	CfBeclin-1	GmAtg6	BmBeclin-1	DpAtg6	CqBeclin-1	AgaBeclin-1	AfBeclin-1	AmAtg6	DqBeclin-1	DaBeclin-1	PpBeclin-1	PxBeclin-1	AaBeclin-1	AtBeclin-1	AdBeclin-1	DmAtg6	AgoBeclin-1	ApBeclin-1	DnBeclin-1	HhBeclin-1	CeBeclin
TmAtg6		89	85	71	68	60	59	59	59	59	59	59	59	59	59	58	58	58	57	56	56	54	54	53	41	29
TcBeclin-1	84		91	76	74	64	63	63	63	64	63	64	64	64	64	64	62	63	62	60	60	59	59	59	44	29
AvBeclin-1	82	92		75	72	62	62	62	62	64	63	63	63	63	62	61	60	63	60	59	60	59	59	58	44	28
AglBeclin-1	70	79	79		79	66	65	64	64	69	68	64	64	64	66	63	64	68	62	64	61	62	62	62	43	32
DvvBeclin-1	67	76	77	84		66	62	62	64	66	63	64	64	65	65	63	62	66	62	60	59	59	59	58	41	33
CfBeclin-1	55	61	61	63	64		67	68	66	70	70	91	91	92	88	69	67	71	68	66	63	63	63	62	47	32
GmAtg6	55	61	60	63	62	65		93	89	68	70	66	66	66	68	92	92	68	93	66	59	60	60	60	43	31
BmBeclin-1	55	61	61	63	62	66	93		89	70	70	68	68	67	69	93	90	70	93	66	60	60	60	59	45	32
DpAtg6	55	61	61	63	63	65	92	91		70	69	66	66	66	67	92	91	71	89	66	60	60	60	59	44	30
CqBeclin-1	55	61	62	66	64	69	65	67	66		86	71	71	70	72	69	68	93	68	80	66	64	64	64	46	34
AgaBeclin-1	56	60	60	63	60	66	64	65	64	83		70	70	68	70	70	69	84	69	89	64	63	63	63	45	33
AfBeclin-1	54	60	61	63	63	91	64	65	64	69	65		99	92	91	68	66	71	67	66	64	63	63	62	46	33
AmAtg6	54	60	61	63	63	92	64	65	64	69	65	100		93	90	68	66	71	67	66	64	63	63	63	47	33
DqBeclin-1	54	61	61	63	64	94	65	65	65	69	65	92	92		87	68	65	71	67	67	63	63	63	62	46	33
DaBeclin-1	54	60	60	63	62	89	66	66	65	70	66	91	91	88		69	67	72	68	67	64	63	63	63	46	32
PpBeclin-1	54	61	61	62	63	66	94	93	93	66	65	65	65	66	67		91	70	93	67	60	60	60	59	44	31
PxBeclin-1	54	60	60	62	61	65	92	90	91	65	64	64	64	64	65	91		68	91	65	60	60	60	60	44	31
AaBeclin-1	54	60	61	64	63	68	64	65	66	93	81	68	68	68	69	66	65		68	79	65	64	64	64	45	33
AtBeclin-1	54	60	60	62	61	66	92	91	89	66	65	64	64	65	66	92	90	65		66	59	59	59	59	43	31
AdBeclin-1	53	57	57	60	58	63	61	62	61	80	88	62	62	63	63	62	61	79	62		60	61	61	61	43	32
DmAtg6	53	59	60	61	61	62	57	57	58	64	62	62	62	62	62	57	58	63	57	60		58	58	58	43	32
AgoBeclin-1	51	58	58	61	59	61	57	57	57	61	58	61	61	61	60	57	58	61	57	57	57		98	98	46	32
ApBeclin-1	51	58	58	62	59	61	57	57	57	61	59	61	61	61	60	57	58	61	57	58	57	98		98	45	32
DnBeclin-1	51	58	58	61	59	61	57	56	57	61	58	61	61	61	60	57	58	61	57	57	56	97	97		45	32
HhBeclin-1	38	40	40	40	39	44	40	41	40	43	42	44	44	43	44	40	40	43	40	40	41	42	42	42		26
CeBeclin	26	28	28	30	30	29	30	30	29	29	28	30	30	30	29	30	29	28	30	27	29	30	30	30	25	

% identity with specific domains

% identity with full-length ORF

Figure S1. Percent identity of TmAtg with its orthologs. The percent identity was determined using specific domain and full length ORF. Maximum identities are circled with green and red in specific domain and full length, respectively.
